# Korean Society of Coloproctology (KSCP) trial of cONsolidation Chemotherapy for Locally advanced mid or low rectal cancer after neoadjUvant concurrent chemoraDiothErapy: a multicenter, randomized controlled trial (KONCLUDE)

**DOI:** 10.1186/s12885-018-4466-7

**Published:** 2018-05-08

**Authors:** Chang Woo Kim, Byung Mo Kang, Ik Yong Kim, Ji Yeon Kim, Sun Jin Park, Won Cheol Park, Ki Beom Bae, Byung-Noe Bae, Seong Kyu Baek, Seung Hyuk Baik, Gyung Mo Son, Yoon Suk Lee, Suk-Hwan Lee

**Affiliations:** 1 Department of Surgery, Kyung Hee University Hospital at Gangdong, Kyung Hee University School of Medicine, 892 Dongnam-ro, Gangdong-gu, Seoul 05278 South Korea; 2 Department of Medicine, the Graduate School of Yonsei University, 50-1 Yonsei-ro, Seoul, South Korea; 3 Department of Surgery, Chuncheon Sacred Heart Hospital, Hallym University College of Medicine, 77 Sakju-ro, Chuncheon, South Korea; 4 Department of Surgery, Wonju Severance Christian Hospital, Yonsei University Wonju College of Medicine, 20 Ilsan-ro, Wonju, South Korea; 5 Department of Surgery, Chungnam National University Hospital, Chungnam National University College of Medicine, 282 Munhwa-ro, Daejeon, South Korea; 6 Department of Surgery, Kyung Hee Medical Center, Kyung Hee University School of Medicine, 23 Kyung Hee dae-ro, Seoul, South Korea; 7 Department of Surgery, Wonkwang University Hospital, Wonkwang University School of Medicine, 895 Muwang-ro, Iksan, South Korea; 8 Department of Surgery, Inje University Busan Paik Hospital, Inje University College of Medicine, 75 Bokji-ro, Busan, South Korea; 9 Department of Surgery, Inje University Sanggye Paik Hospital, Inje University College of Medicine, 1342 Dongil-ro, Seoul, South Korea; 10 Department of Surgery, Keimyung University Dongsan Medical Center, Keimyung University School of Medicine, 56 Dalseong-ro, Daegu, South Korea; 11 Department of Surgery, Gangnam Severance Hospital, Yonsei University College of Medicine, 211 Eonju-ro, Seoul, South Korea; 12 Department of Surgery, Pusan National University Yangsan Hospital, Pusan University College of Medicine, 20 Geumo-ro, Yangsan-si, South Korea; 13 Department of Surgery, Seoul St. Mary’s Hospital, The Catholic University College of Medicine, 222 Banpodae-ro, Seoul, South Korea

**Keywords:** Rectal cancer, Chemoradiotherapy, Consolidation chemotherapy, Pathologic complete response, Disease free survival

## Abstract

**Background:**

Neoadjuvant chemoradiotherapy (CRT) followed by total mesorectal excision (TME) has been a standard treatment option for locally advanced rectal cancer with improved local control. However, systemic recurrence despite neoadjuvant CRT remained unchanged. The only significant prognostic factor proven to be important was pathologic complete response (pCR) after neoadjuvant CRT. Several efforts have been tried to improve survival of patients who treated with neoadjuvant CRT and to achieve more pCR including adding cytotoxic chemotherapeutic agents, chronologic modification of chemotherapy schedule or adding chemotherapy during the perioperative period. Consolidation chemotherapy is adding several cycles of chemotherapy between neoadjuvant CRT and TME. It could increase pCR rate, subsequently could show better oncologic outcomes.

**Methods:**

Patients with advanced mid or low rectal cancer who received neoadjuvant CRT will be included after screening. They will be randomized and assigned to undergo TME followed by 8 cycles of adjuvant chemotherapy (control arm) or receive 3 cycles of consolidation chemotherapy before TME, and receive 5 cycles of adjuvant chemotherapy (experimental arm). The primary endpoints are pCR and 3-year disease-free survival (DFS), and the secondary endpoints are radiotherapy-related complications, R0 resection rate, tumor response rate, surgery-related morbidity, and peripheral neuropathy at 3 year after the surgery. The authors hypothesize that the experimental arm would show a 15% improvement in pCR (15 to 30%) and in 3-year DFS (65 to 80%), compared with the control arm. The accrual period is 2 years and the follow-up period is 3 years. Based on the superiority design, one-sided log-rank test with α-error of 0.025 and a power of 80% was conducted. Allowing for a drop-out rate of 10%, 358 patients (179 per arm) will need to be recruited. Patients will be followed up at every 3 months for 2 years and then every 6 months for 3 years after the last patient has been randomized.

**Discussion:**

KONCLUDE trial aims to investigate whether consolidation chemotherapy shows better pCR and 3-year DFS than adjuvant chemotherapy alone for the patients who received neoadjuvant CRT for locally advanced rectal cancer. This trial is expected to provide evidence to support clear treatment guidelines for patients with locally advanced rectal cancer.

**Trial registration:**

Clinicaltrials.gov NCT02843191 (First posted on July 25, 2016).

## Background

Neoadjuvant chemoradiotherapy (CRT) followed by total mesorectal excision (TME) is the standard treatment for local advanced mid- or low rectal cancer [1, 2]. Reduction of local recurrence has been proven after introduction of TME for rectal cancer [3]. Additionally, chemotherapy and/or radiotherapy have shown the roles to improve oncologic outcomes by controlling local recurrence. Preoperative chemoradiotherapy showed better outcomes compared with postoperative chemoradiotherapy or preoperative radiotherapy alone [4–8].

However, failure of systemic controls including liver, lung, peritoneum, or bone still remained even after CRT followed by TME [1, 4, 5]. Many efforts have been made to control micrometastatic disease and to prevent distant and local recurrence. So far, no reliable molecular or pathologic biomarkers to foster pathologic complete response (pCR), which means complete regression of the tumor after radiotherapy and/or chemotherapy, was identified. Meanwhile, patients who showed pCR have been reported with lower recurrence and better survival [9, 10]. Therefore, many trials have been performed to increase pCR rates by changing perioperative treatment strategies.

Prolongation of the interval between CRT and TME has shown to increase pCR rates [11]. The interval has prolonged recently from 6 to 8 weeks up to 12 weeks, and several authors reported that extending the interval showed better pCR rates without increasing morbidity. [12–15] Another tries to achieve more pCR were adding platinum analogue (i.e. oxaliplatin) or vascular endothelial growth factor antagonist (i.e. bevacizumab) preoperatively and/or postoperatively based on 5-FU [16–18]. Unfortunately most of them failed to show higher pCR rates but increased acute toxicity [19, 20]. Recent trials reported that changing sequence of chemotherapy cycles as well as chemotherapeutic agents would attain better pCR rates, and new trials are launching. The examples are induction chemotherapy, consolidation chemotherapy, and sandwich regimen (prior to, concurrently with, and following radiation therapy) [21–26]. Sandwich regimen with capecitabine and oxaliplatin accompanied by 25 Gy of radiotherapy resulted in 42.2% of pCR rates, and even 10% of patients needed no surgery because the patients had achieved clinical complete response [22]. Garcia-Aguilar et al. reported that pCR rates were significantly increased in the patients who received various cycles of consolidation chemotherapy of FOLFOX regimen without significantly increased toxicity [21]. Then they showed that additional consolidation chemotherapy resulted in higher pCR rates in their multicenter, phase 2 trial [23]. However, it is not sufficient to conclude that consolidation chemotherapy is the only affecting factor to pCR due to several limitations. The interval and duration of consolidation chemotherapy were variable from 6 to 20 weeks. Moreover, adjuvant chemotherapy after TME followed by CRT was not standardized, and the total doses or cycles of chemotherapy was not consistent equally. Therefore, well-designed randomized controlled trials to assess the effect of consolidation chemotherapy alone after neoadjuvant chemoradiotherapy for locally advanced mid or low rectal cancer is required.

## Methods/design

### Study design

This trial is a phase 3 trial investigating the efficacy of 3 cycles of consolidation chemotherapy between neoadjuvant CRT and TME for locally advanced rectal cancer. To prove the effects of consolidation chemotherapy, we will administer the equal schedules of chemotherapy (8 cycles of mFOLFOX6 chemotherapy) in both groups.

Patient selection criteria are as follows:

- Inclusion Criteria

1. An adult aged 20-75 years old
2. Histologically confirmed adenocarcinoma of mid or low rectum (i.e. the lower margin of tumor located below the peritoneal reflection or within 12 cm from the anal verge by digital rectal examination, rigid proctosigmoidoscopy or MRI measurement from sagittal T2 weighted image)
3. Locally advanced rectal cancer confirmed by magnetic resonance image (MRI): Clinical stage T1-3N1or2 or clinical stage cT3N0 (or depth of perirectal invasion by tumor > 5 mm on MRI), or suspicious of circumferential invasion on MRI (or circumferential margin < 1 mm)
4. ECOG performance status of 0-2
5. ASA score of ≤ 3
6. An informed consent form has been signed by the patient.

- Exclusion Criteria

1. Upper rectal cancer (i.e. the lower margin of tumor located above the peritoneal reflection)
2. Clinical stage T1or2N0 on MRI
3. Clinical stage T4Nany on MRI (T4 lesion would be complicated to manage)
4. Distant metastasis diagnosed by imaging diagnosis or histology
5. The patient received chemotherapy or radiotherapy during the past 6 months
6. The patient received any therapy for colorectal cancer or another malignancy during the past 5 years
7. The patient has severe underlying diseases or poor condition to receive chemotherapy or radiotherapy
8. Pregnant of breastfeeding women
9. The patient who participate in another clinical trial, or receives any drug for the trial
10. Uncontrolled peripheral neuropathy (> Grade 2)
11. Any unhealed wound, fracture, peptic ulcer, or intraabdominal abscess
12. Active gastrointestinal bleeding
13. Patients with an active infection, which needs antibiotic therapy, during the randomization period

**Fig. 1:**
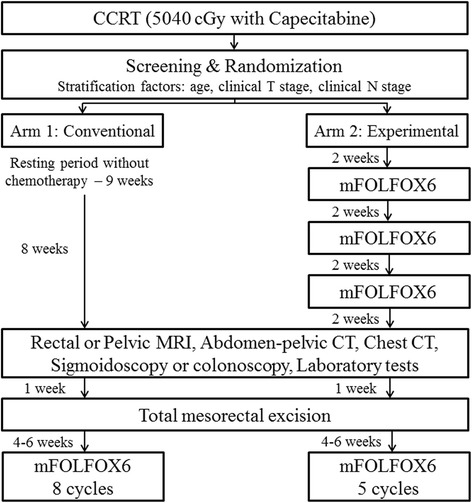
Flowdiagram of the trial

All the patients who received CRT (50.4 Gy radiation with capecitabine 825 mg/m^2^ twice daily for 28 days) will be randomized in a 1:1 ratio into one of the following arms after screening: conventional chemotherapy group (control arm: Arm 1) or consolidation chemotherapy group (experimental arm: Arm 2) (Fig. 1). Stratification factors are age (< 70 or ≥ 70), clinical T stage (T1/2 or T3), and clinical N stage (N0 or N1/2). All eligible patients will be enrolled from approval of institutional review board (IRB) at each participating institution. The accrual period is 2 years and the follow up period is 3 years. The screening program includes history taking, physical examinations, laboratory tests, chest X-ray, EKG, and so on. If abnormal results requiring treatment were found within the screening, the patient will be given a proper treatment and the enrollment can be cancelled.

Arm 1 (conventional chemotherapy group): the patients, who are allocated to the arm 1, will undergo TME at 9 weeks after the CRT. They will receive 8 cycles of adjuvant chemotherapy with mFOLFOX6 (5-fluorouracil with Oxaliplatin) every 2 weeks within 6 weeks after TME. Arm 2 (consolidation chemotherapy group); the patients, who are allocated to the arm 2, will receive 3 cycles of mFOLFOX6 every 2 weeks after neoadjuvant CRT, and undergo TME at 9 weeks after the CRT. Then patients will receive the additional 5 cycles of adjuvant chemotherapy with mFOLFOX6 within 6 weeks after TME.

TME is intended to obtain a complete resection of the tumor (R0) with negative resection margins and adequate lymphadenectomy. The surgeon can decide stoma formation, medial-to-lateral or lateral-to-medial approach, vascular ligation (i.e. high tie or low tie), splenic flexure mobilization as well as the method of operation including open, laparoscopic, or robotic surgery. A complete resection (R0) or microscopic remnant resection (R1) should be confirmed by pathologists after TME. Patient with visible incomplete resection (R2) or distant metastasis is excluded in this study.

mFOLFOX6 regimen is composed of levoleucovorin (200 mg/m^2^) or leucovorin (400 mg/m^2^), oxaliplatin (85 mg/m^2^) for 2 h, then 5-FU (400 mg/m^2^) bolus followed by 5-FU (2400 mg/m^2^) for 46 h. All chemothrapy-related adverse events will be checked every cycle. Patients older than 70 years will receive chemotherapy as 75% reduced dose for the safety. [27–29] When the absolute neutrophil count decreased less than 1500/μl or any drug-related adverse effects of grade 3 were noted, dose reduction or delay of chemotherapy will be considered. However, delay of chemotherapy does not mean delay of TME in Arm 2. Even though a patient in Arm 2 lacks one or two cycles of consolidation chemotherapy due to chemotherapy-related adverse events, the patient will underwent TME at 9 weeks after CRT, and the data will be analyzed as intention-to-treat. All patients will equally visit every 3 months for 2 years, and then every 6 months for 3 years. Physical examination and laboratory test will be performed every 3 months, abdominal-pelvic CT scan every 6 months, chest CT every 1 year, and colonoscopy will be performed at 1 and 5 years to detect metastasis or recurrence.

### Objective

The authors will compare pCR rates and 3-year disease-free survival (DFS) rates as the primary endpoints between the two groups. pCR is defined no residual tumor or complete replacement of fibrosis in the surgical specimen (i.e. Mandard 1 or Dworak 4). 3-yr DFS is defined as proportion of the patients who survive without any local or distant recurrence/metastasis at the 3 years of follow up.

Secondary endpoints are radiotherapy-related toxicity, R0 resection rate, tumor response rate, surgery-related morbidity, and severity of peripheral neuropathy at 3 years. Acute and chronic toxicity will be recorded in the electronic case report form (eCRF). R0 resection rate is defined as the proportion of R0 resection among all TME specimens according to the pathologic results. TME quality evaluation will be performed by surgeons or pathologists: complete, nearly complete, and incomplete [30]. Tumor response rate is represented as grade of Mandard or Dworak [31, 32]. Postoperative complication within 30 days after TME will be recorded according to the grade of Clavien-Dindo classification of surgical complications [33]. Severity of peripheral neuropathy will be assessed at 3 years as well as every cycles of chemotherapy. The grade of any adverse event will be recorded by Common Terminology Criteria for Adverse Events (CTCAE) 4.0 [34]. Operative, pathologic, and chemotherapeutic reports are documented in the eCRF.

### Sample size calculation and randomization

Based on literatures, pCR rates and 3-year DFS after CRT for rectal cancer were known to be 10 to 28% and 50-65%, respectively [2, 6, 8–13]. Thus we hypothesize that the experimental arm would show a 15% improvement in pCR and in 3-year DFS, compared with the control arm (15 to 30% and 65 to 80%, respectively). The accrual period is 2 years and the follow-up period is 3 years. Based on the superiority design, one-sided proportion test and log-rank test with α-error of 0.025 and a power of 90% was conducted for both primary endpoints: pCR and 3-year DFS. The pCR of each group was considered to be 15 and 30%, and the 3-year DFS of each group was considered to be 65 and 80% assuming that accrual time was 2 years and follow up time was 3 years. Allowing neoadjuvant CRT for a drop-out rate of 10%, 358 patients (179 per arm) for pCR and 316 patients (158 per arm) for 3-year DFS will need to be recruited. Therefore a total of 358 participants (among 358 and 316) are needed. This study of 358 participants had at least 81% power (90% each = 0.9, so 0.9 × 0.9 = 0.81) to simultaneously detect a difference of pCR and 3-year DFS of two groups. Randomization will be performed in a 1:1 ratio by computer-generated random numbers, with the use of stratified permuted 4 blocks. It will stratified according to age (< 70 vs. ≥70), clinical T stage (T1/2 or T3), and clinical N stage (N0 or N1/2). Among 8 strata, 2 strata will not be used because clinical T1/2 N0 (age < 70 and ≥ 70) are not indicated for neoadjuvant CRT.

### Data collection and management

**Fig. 2:**
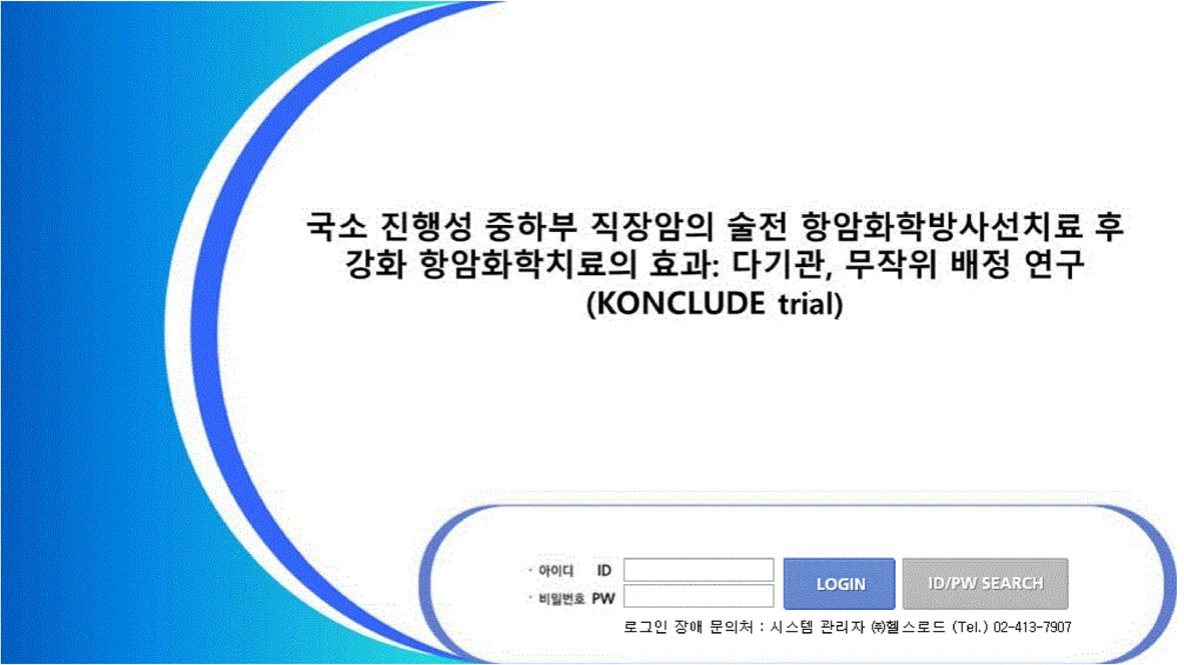
The electronic case report form on line

The authors employ a web-based clinical research management system (Healthroad, Seoul, Republic of Korea), which gathers clinical data and information through on-line (konclude.e-trial.co.kr, Fig. 2). Principal investigators or clinical research coordinators in each site should sign up and enter clinical data onto an eCRF. Approved researchers of central data managing institution will manage the data and information. Only the principal investigator can access the final trial dataset and analyze them. Personal information of enrolled patients will be maintained for 5 years in order to protect confidentiality.

### Drop-out

A patient can be dropped out in cases as follows:

1. Not acceptable adverse events related to surgery or chemotherapy
2. Different treatment needs which not approved in this trial
3. A patient’s refusal by any reasons
4. A patient’s pregnancy

### Statistical analysis

Continuous variables will be analyzed with independent two sample t-test, whereas categorical variables will be analyzed with Chi-square test or Fisher’s exact test. 3-yr DFS will be analyzed with Kaplan-Meier curve. The difference between both Arms will be compared by Cox’s proportional hazards model. Interim analysis for pCR will be performed after TME of the last enrolled patient with Cochran-Mantel-Haenszel test. *P*-value less than 0.05 will be considered as significant. All analyses will be performed with SPSS version 20.0.

### Safety evaluation and reporting of adverse effect

Adverse events and serious adverse events must be reported to protect patients. Radiotherapy-related toxicity, chemotherapy-related toxicity, and surgery-related morbidity will be recorded onto the eCRF. Serious adverse events will be reported every quarter of the year, whereas suspected, unexpected, serious, adverse reaction, which could result in death or life threatening, will be reported within 15 days from the detection by investigators.

### Data monitoring

A committee will be organized for trial supervision. All members of the committee are certified according to the course of good clinical practice in each institution. All process including data collection, record, and management will be monitored by them. The committee can advise and request the principal investigator to change some plans.

### Protocol modification

If needed, the protocol can be modified by communication and agreement of the principal investigator and trial participants with revising its version.

## Discussion

Despite fundamental role of 5-FU as neoadjuvant and adjuvant regimen for advanced colorectal cancer, addition of various cytotoxic agents has been tried to improve oncologic outcomes. Among them was oxaliplatin, a platinum analogue (Table 1). FOLFOX as adjuvant regimen for locally advanced rectal cancer showed superior DFS compared with 5-FU or capecitabine alone in ADORE trial and the German CAO/ARO/AIO-04 study [35, 36], although it failed to show better oncologic outcomes but more proportion of acute toxicity as neoadjuvant regimen [7, 17, 18]. Surgical and medical oncologists concluded that FOLFOX is only efficient as adjuvant setting, not neoadjuvant for rectal cancer. Then, can FOLFOX show higher pCR rates without increasing acute toxicity in the position between neoadjuvant and adjuvant? This is the point that KONCLUDE trial concentrates on.

**Table 1:** pCR by adding Oxaliplatin to 5-FU for rectal cancer

Trial	*N*	Regimen	pCR rate
STAR-01	705	5-FU vs. 5-FU + Oxaliplatin	16% vs. 16% (*p* = 0.904)
ACCORD 12	598	Capecitabine vs. CAPOX	13.9% vs. 19.2% (*p* = 0.09)
NSABP R-04	1608	5-FU vs. 5-FU + Oxaliplatin vs. Capecitabine vs. CAPOX	17.8% vs. 19.5% (*p* = 0.42)
CAO/ARO/AIO-04	1236	5-FU vs. 5-FU + Oxaliplatin	13% vs. 17% (*p* = 0.031)
PETACC-6	1094	Capecitabine vs. CAPOX	11.3% vs. 13.3% (*p* = 0.31)
Dellas et al.	70	CAPOX + Bevacizumab	17.4%
EXPERT-C	165	CAPOX vs CAPOX + Cetuximab	7% vs. 11% (*p* = 0.714)

*pCR* pathologic complete response, *5-FU* 5-fluorouracil, *CAPOX* capecitabine with oxaliplatin

As aforementioned, several authors already reported high pCR rates from consolidation chemotherapy without significantly increasing toxicity, while a couple of trials are on-going [21–25]. However, there were a few, but meaningful limitations in their studies including characters of phase 2 trial, variation of the resting period between radiotherapy and TME, and difference of total cycles of chemotherapy. These studies cannot establish the treatment guideline because of their inherited bias. Therefore, we adjusted total cycles of chemotherapy as 8 cycles of mFOLFOX6 to avoid inherited bias due to different cycles of chemotherapy. Moreover, we fixed the interval between radiotherapy and TME as 9 weeks, according to previous literatures which commented appropriate resting period as 6 to 12 weeks, to investigate efficacy of consolidation without confounding of various duration of interval from completion of CRT to TME.

KONCLUDE trial started with the first enrollment in December 7, 2016 after the registration on clinicaltrials.gov in July 25, 2016 (NCT02843191). We expect that this trial will conclude whether consolidation chemotherapy would attain higher pCR and improved DFS or not, providing a critical clue to establish the standard treatment in locally advanced mid- or low rectal cancer with level 1 evidence.

## Abbreviations

5-FU: 5-fluorouracil
CRT: chemoradiotherapy
DFS: disease-free survival
eCRF: electronic case report form
FOLFOX: 5-FU and leucovorin with oxaliplatin
KONCLUDE: Korean society of coloproctology trial of cONsolidation Chemotherapy for Locally advanced mid or low rectal cancer after neoadjUvant concurrent chemoraDiothErapy: a multicenter, randomized controlled trial
pCR: pathologic complete response
TME: total mesorectal excision
